# Congenital syndromic Chiari-like malformation (CSCM) in Holstein cattle: towards unravelling of possible genetic causes

**DOI:** 10.1186/s13028-024-00752-y

**Published:** 2024-07-04

**Authors:** Joana Goncalves Pontes Jacinto, Anna Letko, Irene Monika Häfliger, Cord Drögemüller, Jørgen Steen Agerholm

**Affiliations:** 1 Institute of Genetics, Vetsuisse Faculty, University of Bern, Bremgartenstrasse 109a, Bern, 3012 Switzerland; 2 Clinic for Ruminants, Vetsuisse Faculty, University of Bern, Bremgartenstrasse 109a, Bern, 3012 Switzerland; 3 Department of Veterinary Clinical Sciences, University of Copenhagen, Højbakkegaard Allé 5A, Taastrup, 2630 Denmark

**Keywords:** *Bos taurus*, Chromosomal abnormalities, Congenital defect, *DYNC1H1*, Haploinsufficiency, Neural tube defect, Precision medicine, Rare disease, *SHC4*, Spina Bifida, *WDR45B*

## Abstract

**Background:**

Chiari malformation type II (CMII) was originally reported in humans as a rare disorder characterized by the downward herniation of the hindbrain and towering cerebellum. The congenital brain malformation is usually accompanied by spina bifida, a congenital spinal anomaly resulting from incomplete closure of the dorsal aspect of the spinal neural tube, and occasionally by other lesions. A similar disorder has been reported in several animal species, including cattle, particularly as a congenital syndrome. A cause of congenital syndromic Chiari-like malformation (CSCM) in cattle has not been reported to date. We collected a series of 14 CSCM-affected Holstein calves (13 purebred, one Red Danish Dairy F1 cross) and performed whole-genome sequencing (WGS). WGS was performed on 33 cattle, including eight cases with parents (trio-based; group 1), three cases with one parent (group 2), and three single cases (solo-based; group 3).

**Results:**

Sequencing-based genome-wide association study of the 13 Holstein calves with CSCM and 166 controls revealed no significantly associated genome region. Assuming a single Holstein breed-specific recessive allele, no region of shared homozygosity was detected suggesting heterogeneity. Subsequent filtering for protein-changing variants that were only homozygous in the genomes of the individual cases allowed the identification of two missense variants affecting different genes, *SHC4* in case 4 in group 1 and *WDR45B* in case 13 in group 3. Furthermore, these two variants were only observed in Holstein cattle when querying WGS data of > 5,100 animals. Alternatively, potential *de novo* mutational events were assessed in each case. Filtering for heterozygous private protein-changing variants identified one *DYNC1H1* frameshift variant as a candidate causal dominant acting allele in case 12 in group 3. Finally, the presence of larger structural DNA variants and chromosomal abnormalities was investigated in all cases. Depth of coverage analysis revealed two different partial monosomies of chromosome 2 segments in cases 1 and 7 in group 1 and a trisomy of chromosome 12 in the *WDR45B* homozygous case 13 in group 3.

**Conclusions:**

This study presents for the first time a detailed genomic evaluation of CSCM in Holstein cattle and suggests an unexpected genetic and allelic heterogeneity considering the mode of inheritance, as well as the type of variant. For the first time, we propose candidate causal variants that may explain bovine CSCM in a certain proportion of affected calves. We present cattle as a large animal model for human CMII and propose new genes and genomic variants as possible causes for related diseases in both animals and humans.

**Supplementary Information:**

The online version contains supplementary material available at 10.1186/s13028-024-00752-y.

## Background

Congenital neural tube defects (NTDs) develop during embryogenesis as a result of a failure of the morphogenic process of neural closure [1]. They are the most severe congenital malformations of the central nervous system (CNS) and among the most frequent congenital defects in humans [2]. Common NTDs are phenotypically classified into anencephaly, myelomeningocele (spina bifida), craniorachischisis, and encephalocele with respect to the anatomical location of the defect [3]. Both genetic and non-genetic factors such as environmental factors appear to be involved in the aetiology of human NTDs [1, 4]. In humans, deleterious variants in the *VANGL1*, *VANGL2*, *TBXT*, *CCL2* and *FUZ* have been associated with susceptibility to develop NTDs (OMIM 182,940). Moreover, pathogenic variants in *MTHFR*, *MTR*, *MTRR* and *MTHFD1* have been associated with folate-sensitive NTDs (OMIM 601,634). Chromosomal abnormalities have also been linked with NTDs in human medicine; however, they represent only 2–16% of isolated NTDs [5]. In addition to genetic causes, it has been suggested that the maternal folate status is highly associated with the development of NTDs in children [6].

Chiari malformation (CM) was described in human medicine over a century ago [7] and represents a heterogeneous group of anomalies of the posterior fossa and hindbrain (cerebellum, pons, and medulla oblongata) subdivided in four types (Chiari I-IV) [8–10]. As main findings, herniation of the cerebellar tonsils through the foramen magnum, or absence of the cerebellum with or without other associated intracranial or extracranial defects (e.g., hydrocephalus, syrinx, encephalocele or spinal dysraphism), may be observed [11–13].

In cattle, sporadic cases of a congenital syndrome resembling human CM type II (CMII) have been reported for more than 70 years in various breeds without recognition of a familial pattern [14–17]. Affected calves showing a congenital syndromic Chiari-like malformation (CSCM) have been characterized by concurrent spina bifida and hindlimb arthrogryposis. A different rare bovine syndrome also seems to exist in which CSCM is accompanied by dicephaly [18, 19]. Possible causes of CSCM in cattle have yet not been reported.

The aim of this study was to genomically evaluate a series of CSCM-affected calves and, where available, their parents, by whole-genome sequencing (WGS) to assess whether CSCM in cattle is associated with genetic variants.

## Methods

### Cattle and phenotypical investigation

Eleven calves submitted to the Department of Veterinary Clinical Sciences, University of Copenhagen, Denmark from 2016 to 2020 and diagnosed with CSCM by necropsy were included in this study. Seven cases were stillbirths, and four were born alive but euthanized due to poor prognosis and welfare reasons. In addition, material from four suspected CSCM cases was also included. The latter cases were assessed based on photographs taken by the herd veterinarian who performed the on-farm sampling. Biological materials were stored at -20 °C until being analysed. For DNA analysis, the cases were divided into three groups: group 1 consisted of the cases in which tissue was available from the CSCM-affected calf and both parents (trios: case-parents) (*n* = 8), group 2 consisted of the cases in which tissue was available from the CSCM-affected calf and only one parent (duos: case-parent) (*n* = 3), and group 3 consisted of cases in which only tissue from the affected calf was available (solo: case only) (*n* = 3). One case was an F1 cross, Red Danish Dairy x Holstein (case 1), while the remaining cases (cases 2–14) were purebred Holstein (*n* = 13). Four CSCM-affected calves were female and ten were male (Additional file 1). Three-generation pedigrees of the parents were obtained from the Danish Cattle Database and analysed for inbreeding loops and common ancestors across the case series. Extended pedigrees were analysed when recessive candidate variants were identified.

### DNA extraction

Genomic DNA was extracted from ear cartilage from all CSCM cases and their parents when available (EDTA-stabilized blood from dams and semen from sires) using the Maxwell RSC DNA System (Promega).

### Whole-genome sequencing and variant calling

WGS data were generated for a total of 33 animals using the Illumina NovaSeq6000 (Illumina Inc.). Trio-based approach was performed for 24 animals from group 1, the single parent-based approach was used for six animals from group 2 and the solo-based approach was used for the three individual cases from group 3. Sequenced reads were aligned to the ARS**-**UCD1.2 reference genome [20]. The average coverage obtained for each genome is shown in Additional file 1. Single-nucleotide variants (SNVs) and small indel variants were called. The applied software and steps to process fastq files into binary alignment map and genomic variant call format files followed the 1000 Bull Genomes Project (run 9) [21], except for trimming, which was performed using fastp [22]. Further processing of the genomic data was performed as previously reported [23]. To find private variants, we compared the genotypes of the CSCM case(s) with 1,023 bovine genomes of different breeds sequenced as part of the ongoing Swiss Comparative Bovine Resequencing Project. Regarding the mode of inheritance, three different scenarios were hypothesized: (i) autosomal recessive mode of inheritance common to all purebred Holstein CSCM cases, (ii) autosomal recessive mode of inheritance considering each CSCM case individually, or (iii) dominant mode of inheritance acting *de novo* considering each CSCM case as an isolated event due to spontaneous mutations. Integrative Genomics Viewer (IGV) [24] was used for visual assessment of genomic regions containing potential candidate genes.

### Sequencing-based genome-wide association study and homozygosity analysis

A sequencing-based genome-wide association study (seqGWAS) was performed in order to test the hypothesis of a causal recessive variant common to all Holstein CSCM cases. Biallelic variants were selected from the vcf file using PLINK v1.9 [25] as a common quality control step. For the control cohort, 166 phenotypically normal, not closely related Holstein cattle (identical by descent < 0.5 as detected by PLINK) [25] were selected from the Swiss Comparative Bovine Resequencing project. In addition, a genome-wide search for homozygous regions shared by the Holstein cases was performed using the R package detectRUNS v.0.9.6 [26].

### Investigation of copy number variation

To assess possible larger structural variants and chromosomal abnormalities, including chromosomal, numerical, and structural abnormalities, the depth of coverage along all chromosomes was calculated. A sliding window approach was used with two different window sizes (10 kb, 200 kb). The bedcov function of Samtools [27] was used to generate the number of reads within each specified window. Coverage plots were generated using the Manhattan function of the qqman package in R [28].

### Occurrence of variants in a global control cohort

The comprehensive variant catalogue from run 9 of the 1000 Bull Genomes Project was available to investigate the allelic distribution of variants within a global control cohort [21]. The full dataset includes 5,116 bovine genomes, including 576 from the Swiss Comparative Bovine Resequencing Project, from a wide variety of more than 130 breeds. There were 1,209 Holstein and 74 Red Danish Dairy cattle in the dataset. This allowed the exclusion of variants common to these breeds.

### In silico assessment of the molecular consequences

PredictSNP1 [29] and/or Provean [30] were used to predict the biological consequences of the candidate protein-changing variants. The gnomAD browser was used to predict the probability of the orthologue human gene being loss-of-function intolerant (pLI score) [31].

### Candidate gene and candidate variant definitions

The term “candidate gene” was used to describe genes based on function and/or associated phenotype in mammalian species. The term “candidate variant” was used to describe variants considering the affected gene function and/or associated phenotype in mammalian species, rarity, and the predicted effect of the variant on the encoded protein. All sequence accessions used for the candidate variants are listed in Additional file 2.

### Comparative chromosomal alignment

The Comparative Genome Viewer [32] was used to compare two genomes based on assembly-assembly alignments provided by National Center for Biotechnology Information (NCBI), forbovine chromosomes 2 and 12, between *Bos taurus* assembly ARS-UCD2.0 (GCF_002263795.3) and *Homo sapiens* assembly GRCh38.p14 (GCF_000001405.40).

## Results

### Phenotypical features of bovine CSCM

The body weight (BW) was bellow average in all cases. One case (case 1, Red Danish x Holstein) had a BW of only 7.3 kg despite being delivered on gestation day 272. The BW of the other cases (Holstein) ranged from 26.0 to 37.3 kg (mean = 30.6 kg; SD = ± 3.3) compared to the reported average birth weight of 42 kg for Holstein calves in Denmark [33]. The gestation period of Holstein dams ranged from 266 to 296 days (mean = 281 days; SD = ± 8.5) which is within the reference range for Holsteins in Denmark [33].

**Fig. 1:**
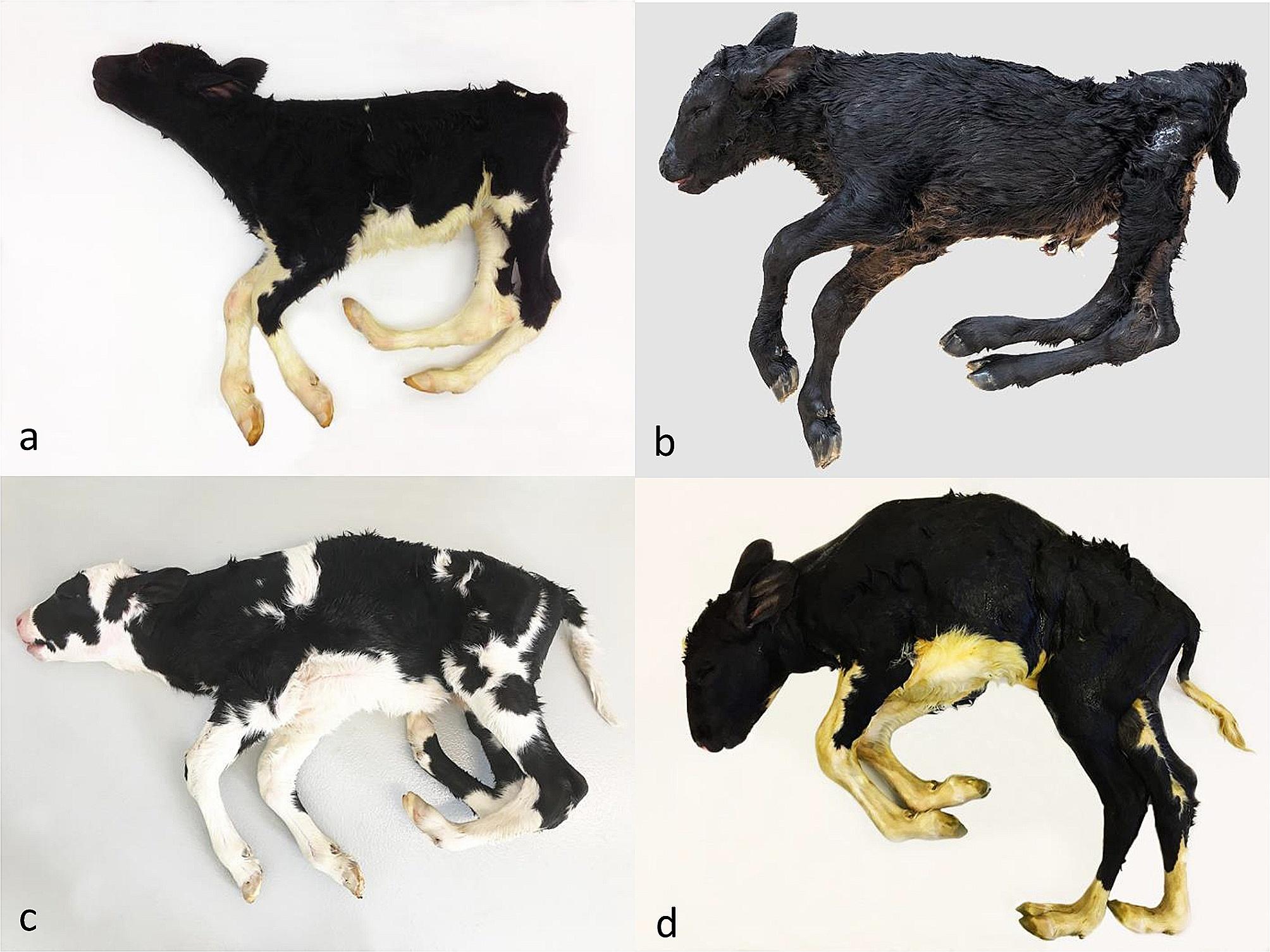
External appearance of four cases of congenital syndromic Chiari-like malformation (CSCM) in cattle. Although a certain morphological variation is seen, the cases present a shared overall syndromic lesion prototype being characterized by hind quarter hypoplasia, bilateral almost symmetrical arthrogryposis of the hip, stifle, tarsal and fetlock joints with normally developed front limbs, and flattening of the neurocranium. The calves shown in **a-d** represents cases 2, 8, 11 and 13, respectively

**Fig. 2:**
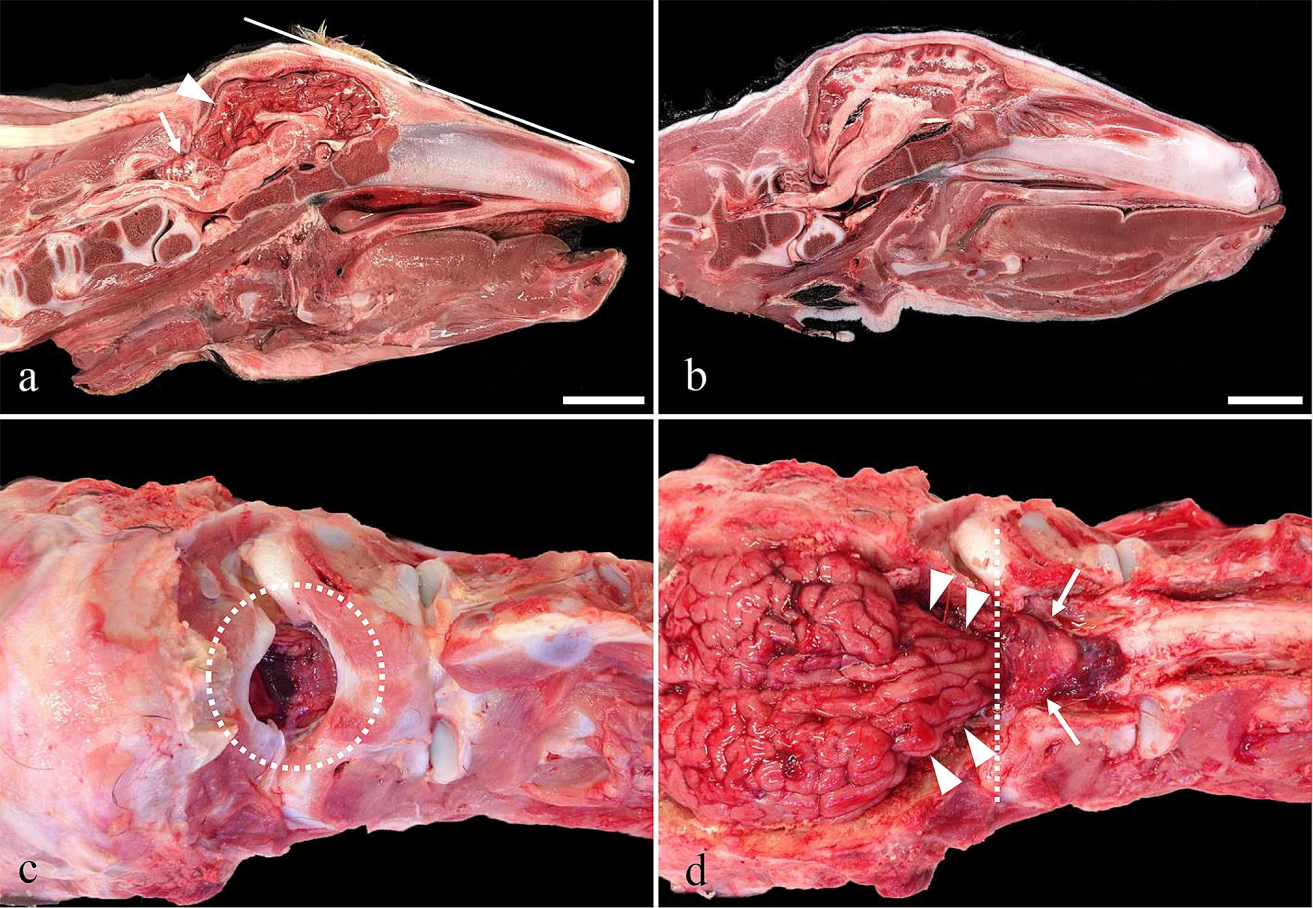
Head and brain malformation in CSCM in cattle. **a**) Flattening of the neurocranium as indicated by a straight line causes caudal displacement and compression of the occipital lobes (arrowhead), midbrain, and cerebellum. Partial herniation of the medulla oblongata and cerebellum into the first segments of the cervical vertebral canal is seen (arrow). Case 6, longitudinal midplane section. **b**) Same as a) but the cerebellum has been completely displaced into the vertebral canal and caudal elongation of the occipital lobes is pronounced. Dotted line: foramen magnum. Case 8, longitudinal midplane section. **c**) Dorsal view towards the atlantooccipital region after removal of the skin, muscles, connective tissue, and dura mater. The cerebellum (encircled) being displaced through the foramen magnum can be seen in the vertebral canal of the first cervical vertebra. Dotted line: foramen magnum. Case 12. **d**) Same as c) after parts of the neurocranium and the vertebral arches have been removed. Bilateral symmetrical displacement and elongation of the occipital lopes is evident and is the extracranial displacement of the cerebellum. Case 12

The necropsied cases presented a common overall syndromic lesion prototype. The external appearance was characterized by hypoplasia of the hindlimbs and bilateral almost symmetrical arthrogryposis of the hip, stifle, tarsal and fetlock joints, whereas the forelimbs were normally developed (Fig. 1). The hip, stifle and fetlock joints were usually extended, while the tarsal joints were flexed up to 90°. Anchylosis of the hip, stifle and fetlock joints was common. The pelvic region and hindlimb muscles were hypoplastic with variable degrees of lipomatous muscular dysplasia. Head examination revealed a marked flattening of the neurocranium. An almost straight line could be drawn across the dorsomedial sagittal plane of the viscerocranium over the anterior part of the neurocranium (Fig. 2a). The flattening of the neurocranium was associated with compression of the brain hemispheres and caudal displacement of the occipital lobes, midbrain, and cerebellum causing either partial or complete herniation of the medulla oblongata and cerebellum into the first segments of the cervical vertebral canal and elongation of the occipital lobes (Fig. 2).

**Fig. 3:**
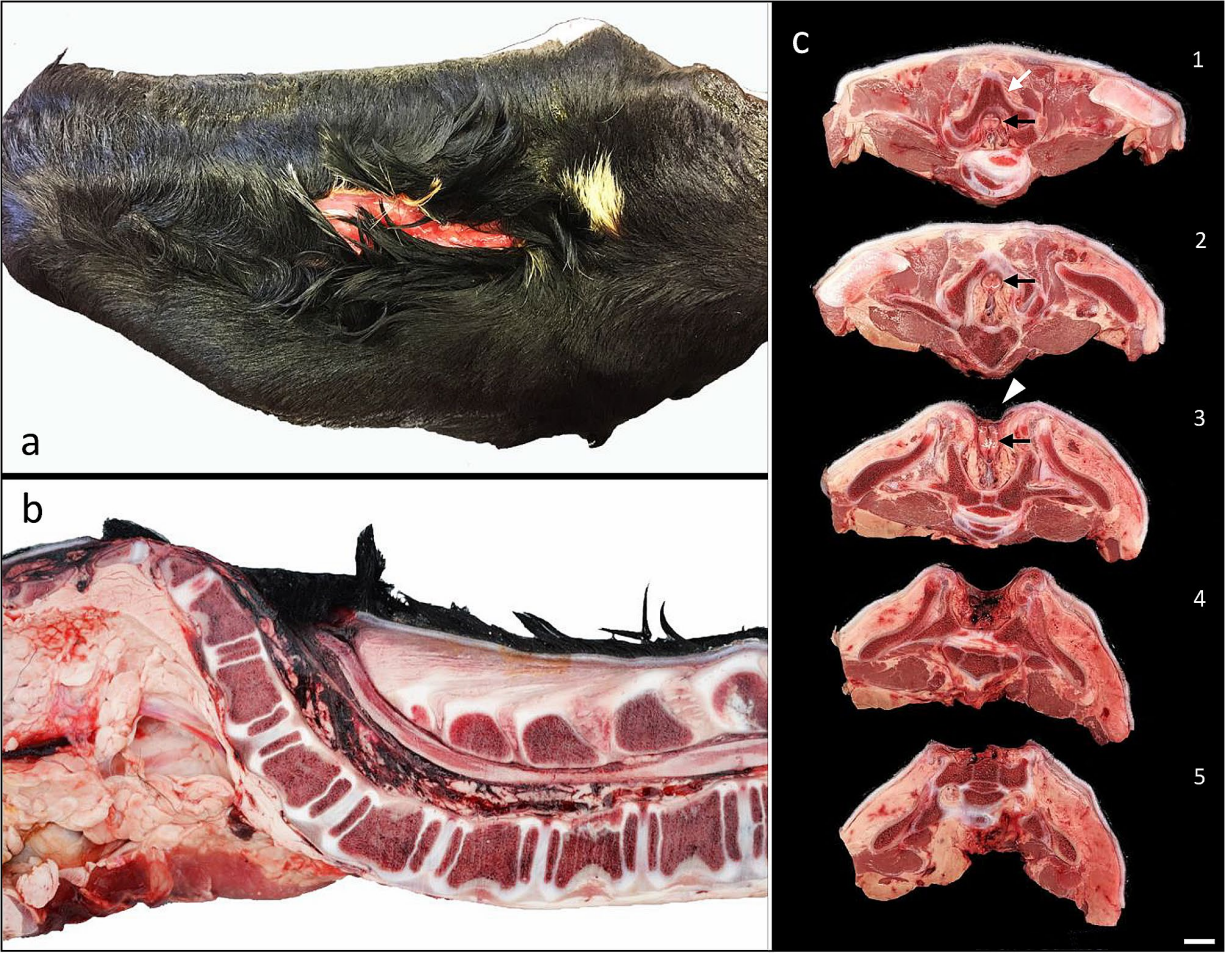
Spina bifida associated with CSCM in cattle. **a**) A cranio-caudal elongated pink hairless lesion (sulcus) is present in the dorsal midline of the most caudal lumbar vertebrae, typically with a length of 5 cm and a width of 1.5 cm with some variation among cases. **b**) Longitudinal midplane section of the lumbar and sacral spine showing absence of the vertebral arches of the most caudal lumbar vertebrae and deviation of the course of the spine (kyphosis). **c**). Serial transverse sections in cranio-caudal direction (Sects. 1–5) of the most caudal segments of the lumbar spine. The spinal cord is indicated by black arrows, the vertebral arch by a white arrow and sulcus by a white arrowhead

Examination of the lumbosacral spine revealed spina bifida in all cases. A pink hairless lesion (sulcus) was present in the dorsal midline of the most caudal lumbar/most cranial sacral vertebrae, typically cranio-caudally elongated with a length of 5 cm and a width of 1.5 cm with some variation between cases (Fig. 3a). Longitudinal mid-plane or serial transverse sawing of the lumbar and sacral spines (Fig. 3b, c) revealed absence of the arches of the most caudal lumbar vertebrae and deviation of the course of the spine, usually kyphosis. The most caudal segments of the lumbar spinal cord segments, epidural fat, and the cauda equina were exposed at the base of the sulcus (Fig. 3). Meningeal or spinal cord herniation above the skin surface was not observed.

A number of additional malformations were identified, but none of these were present in all cases. The most common malformations were renal anomalies (unilateral agenesis, hydronephrosis, fused kidneys), cryptorchidism, and intestinal atresia. The general necropsy findings are shown in Table 1.

**Table 1:** Overview of the included cases of congenital syndromic Chiari-like malformation (CSCM) in cattle

Case ID	Material	Breed	Sex	BW (kg)	GA (days)	Gross morphology*
1	Carcass	Red Danish x Holstein	Female	7.3	272	Proportional growth retardation; reduced prominence of the calvarium associated with caudal dislocation of the brain and partial herniation of the cerebellum and medulla oblongata through the foramen magnum (CMII); lumbar spina bifida, lack of vertebral arches, lordosis and lumbar meningocele; bilateral symmetrical extension of the stifle joints and flexion of the tarsal joints; dysplasia (lipomatous) of the hindquarter muscles; agenesis of the right kidney
2	Carcass	Holstein	Female	30.5	278	Reduced prominence of the calvarium associated with caudal dislocation of the brain and partial herniation of the cerebellum and medulla oblongata through the foramen magnum (CMII); lumbar spina bifida and lack of vertebral arches; coccygeal agenesis; bilateral symmetrical extension of the stifle and fetlock joints, flexion of the tarsal joints; hypoplasia and dysplasia (lipomatous) of the hindquarter muscles; agenesis of the right kidney and caudal displacement of the left kidney; anal atresia
3	Carcass	Holstein	Male	32.0	277	Reduced prominence of the calvarium associated with caudal dislocation of the brain and partial herniation of the cerebellum and medulla oblongata through the foramen magnum (CMII); hydrocephalus and dilation of the mesencephalic duct; thoracic kyphosis with fusion of ribs nos. 8 and 9; reduced volume of the thorax; pulmonary hypoplasia and misshapen hearth; lumbar spina bifida, lack of vertebral arches and lumbar kyphosis; coccygeal agenesis; bilateral symmetrical extension of the stifle, tarsal and fetlock joints; hypoplasia and dysplasia (lipomatous) of the hindquarter muscles; anal atresia; fusion of the kidneys at their caudal pole (“horse-shoe” shaped kidneys); bilateral abdominal cryptorchidism
4	Tissue	Holstein	Male	NR	280	Photo assessment: Lumbar spina bifida; bilateral symmetrical flexion of the stifle and tarsal joints. Head morphology not assessable
5	Tissue	Holstein	Male	NR	285	Photo assessment: Lumbar spina bifida. Head and limb morphology not assessable
6	Carcass	Holstein	Male	32.7	272	Reduced prominence of the calvarium associated with caudal dislocation of the brain and partial herniation of the cerebellum and medulla oblongata through the foramen magnum (CMII); lumbar spina bifida, lack of vertebral arches and lumbar lordosis; coccygeal scoliosis; Asymmetrical arthrogryposis of the hind limbs (left: extension of stifle and fetlock joints and flexion of the tarsal joints; right: flexion of the stifle, tarsal and fetlock joints with tarsal anchyloses); dysplasia (lipomatous) of the hindquarter muscles; bilateral abdominal cryptorchidism
7	Carcass	Holstein	Female	27.4	283	Reduced prominence of the calvarium associated with caudal dislocation of the brain and herniation of the cerebellum and medulla oblongata through the foramen magnum (CMII); lumbar spina bifida, lack of vertebral arches and lumbar lordosis and scoliosis; bilateral symmetrical flexion of the stifle and tarsal joints; hypoplasia of the hindquarter muscles with occasional lipomatous dysplasia
8	Carcass	Holstein	Male	37.3	NR	Reduced prominence of the calvarium associated with caudal dislocation of the brain and partial herniation of the cerebellum and medulla oblongata through the foramen magnum (CMII); lumbar spina bifida, lack of vertebral arches and lumbar lordosis; bilateral symmetrical extension of the stifle and flexion of the tarsal joints; dysplasia (lipomatous) of the hindquarter muscles; fusion of the kidneys at their caudal pole (“horse-shoe” shaped kidneys), unilateral hydronephrosis and a shared (hydro-) ureter; abdominal cryptorchidism and fused epididymis; rectal and anal atresia
9	Tissue	Holstein	Male	NR	296	Photo assessment: Lumbar spina bifida; bilateral symmetrical flexion of the stifle and tarsal joints, extension of the fetlock joints. Head morphology not assessable
10	Carcass	Holstein	Female	30.0	288	Reduced prominence of the calvarium associated with caudal dislocation of the brain and herniation of the cerebellum and medulla oblongata through the foramen magnum (CMII); hydrocephalus and dilation of the mesencephalic duct; cervical scoliosis; lumbar spina bifida, lack of vertebral arches and lumbar kyphosis; bilateral symmetrical extension of the stifle and flexion of the tarsal joints; dysplasia (lipomatous) of the hindquarter muscles. Triplets – only one malformed (and examined).
11	Carcass	Holstein	Male	27.1	266	Reduced prominence of the calvarium associated with caudal dislocation of the brain and partial herniation of the cerebellum and medulla oblongata through the foramen magnum (CMII); lumbar spina bifida, lack of vertebral arches and lumbar lordosis; bilateral symmetrical flexion of the tarsal joint and extension of the fetlock joints; dysplasia (lipomatous) of the hindquarter muscles
12	Carcass	Holstein	Male	32.5	276	Reduced prominence of the calvarium associated with caudal dislocation of the brain and partial herniation of the cerebellum and medulla oblongata through the foramen magnum (CMII); lumbar spina bifida, lack of vertebral arches and lumbar kyphosis; bilateral symmetrical extension of the stifle, tarsal and fetlock joints with anchyloses of the stifle; dysplasia (lipomatous) of the hind quarter muscles; subaortal interventricular septal defect, dilation of the left ventricle and right sided myocardial hypertrophy; abdominal (right) and inguinal (left) cryptorchidism, hypoplasia of the left kidney
13	Carcass	Holstein	Male	26.0	295	Reduced prominence of the calvarium associated with caudal dislocation of the brain and partial herniation of the cerebellum and medulla oblongata through the foramen magnum (CMII), hypophyseal fossa not developed, thoracic scoliosis, lumbar spina bifida, lack of vertebral arches and lumbar kyphoscoliosis, bilateral symmetric flexion of the carpal joints, bilateral symmetric extension of all hind limb joints with anchylosis, dysplasia of the hind quarter muscles, bilateral inguinal cryptorchidism
14	Tissue	Holstein	Male	NR	278	Photo assessment: Lumbar spina bifida; bilateral symmetrical flexion of the stifle and tarsal joints, extension of the fetlock joints. Head morphology not assessable

* The brain was examined by transverse sectioning in five cases (Cases 1, 3, 10, 12 and 13) and by midplane longitudinal sawing of the entire head in the remaining cases. BW: body weight; GA: gestation age; NR: not recorded

Evaluation of the three photo documented cases revealed the presence of lumbosacral spina bifida, hypoplasia of the hindlimbs and bilateral, almost symmetrical arthrogryposis of the hip, stifle, tarsal and fetlock joints with normally developed forelimbs. The images did not allow the definitive diagnosis of herniation of the hindbrain, but the neurocranium appeared to be flattened.

### Evidence for independent rare candidate causal recessive CSCM alleles in Holstein cattle

Assuming a simple recessive mode of inheritance, the WGS data were filtered for homozygous coding variants privately present in the 13 CSCM-affected Holstein calves, but without identifying any single-nucleotide or small indel variants common to all cases. Moreover, for the Holstein CSCM cases, the seqGWAS results revealed 13 statistically significant single variants, but no clearly associated genomic region (above the Bonferroni threshold, *P*-value < 3.2 × 10^− 09^; Additional files 3 and 4). All of these polymorphic sites were further visualized in IGV, revealing that the identified variants were most likely artefacts also present in unrelated, unaffected cattle from the global control cohort. In addition, the seqGWAS was also performed excluding the two Holstein cases 1 and 7 where a structural variant was identified (Additional files 3 and 4) but similarly no clearly associated genomic region was identified. Three autosomal regions of shared homozygosity were detected in 11 cases. These results suggest that a single common recessive allele is unlikely to explain the development of CSCM in Holstein cattle.

**Fig. 4:**
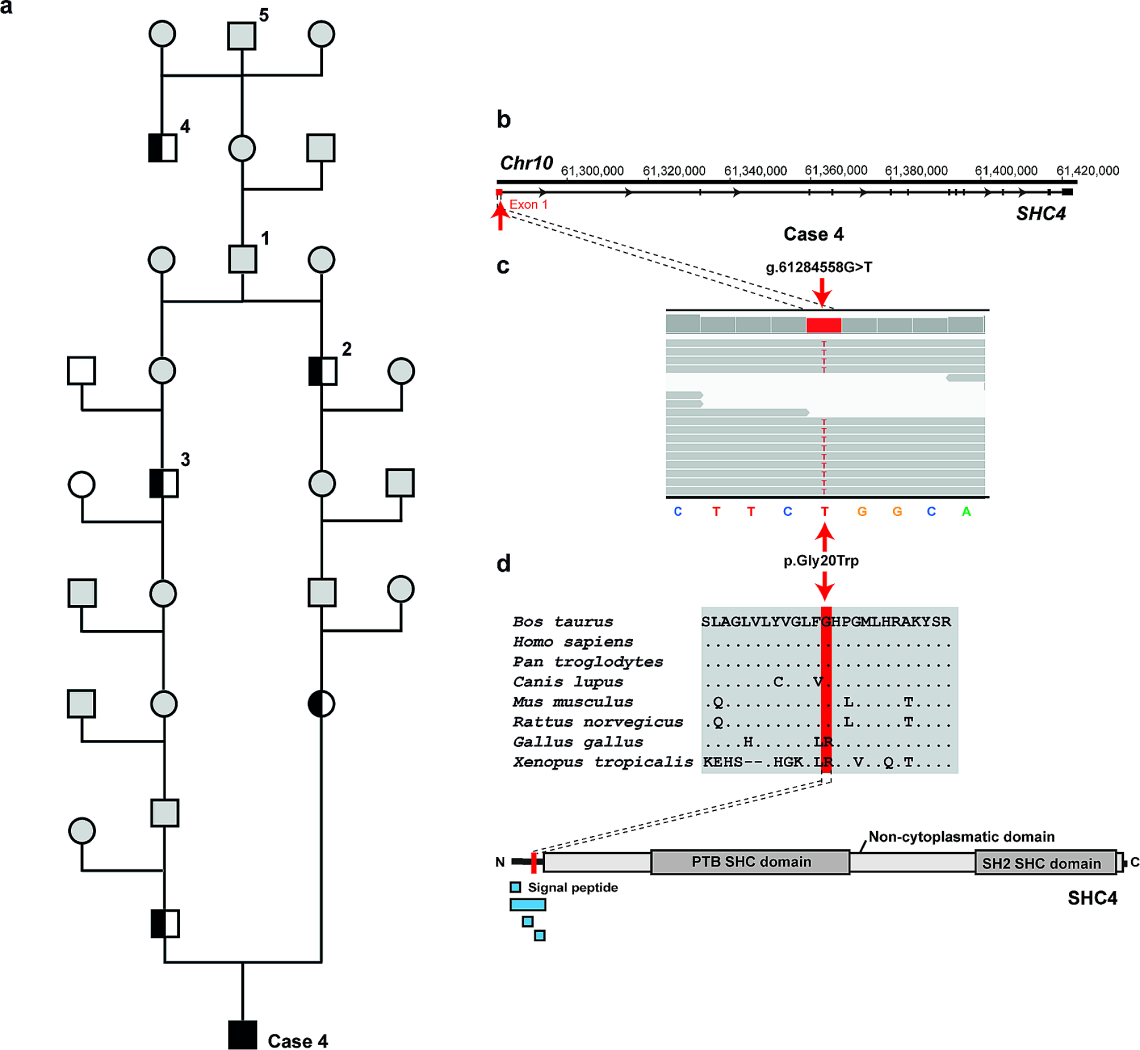
A case of bovine CSCM with a homozygous *SHC4* missense variant. **a**) Case 4: Pedigree for the *SHC4-*related CSCM in case 4. The affected calf is the result of breeding between heterozygous parents (sire Cape P and cow 1793). Both parents can be genetically traced to the common ancestor Kinglea Leader (1). Genotyping of animals included in the 1000 Bull Genomes Project revealed that the sires Kommandeur Leader 1 (2), Ladino Park Talent (3), and Bit-O-Wind Starwar (4) were heterozygous for the *SHC4* variant. Case 4 and diagnosed carriers in its pedigree were genetically related to Bit-O-Wind Starwar through the sire Wapa Arlinda Conductor (5). Black square: homozygous mutant male; half-black square: heterozygous male; white square: homozygous wild-type male; white circle: homozygous wild-type female; grey circle: female unknown genotype; grey square: male unknown genotype. **b**) *SHC4* gene structure showing the location of the homozygous missense variant on chromosome 10, exon 1 (red arrow). (**c**) IGV screenshot showing the Chr10:g.61284558G > T variant homozygous in the affected calf revealed by whole-genome sequencing (shown below). **d**) Multiple sequence alignment of the SHC4 protein encompassing the region of the p.Gly20Trp variant reveals complete evolutionary conservation across mammals. The schematic representation of the bovine SHC4 protein and its functional domains is shown below. The position of the p.Gly20Trp variant is indicated in red

**Fig. 5:**
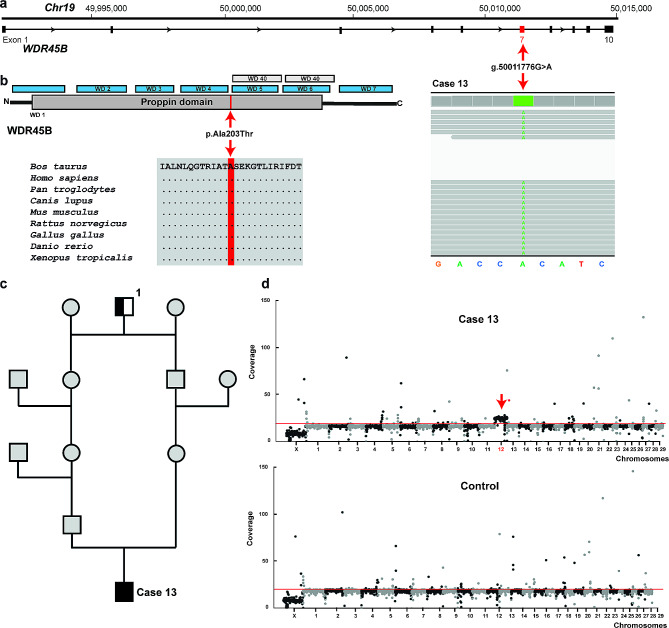
A case of bovine CSCM with a homozygous *WDR45B* missense variant and trisomy 12. **a**) *WDR45B* gene structure showing the location of the homozygous missense variant on chromosome 19, exon 7 (red arrow). IGV screenshot showing the Chr19:g.50011776G > A variant homozygous in the affected calf revealed by whole-genome sequencing (shown below). **b**) The schematic representation of the bovine WDR45B protein and its functional domains. The position of the p.Ala203Thr variant is indicated in red. Multiple sequence alignment of the WDR45B protein encompassing the region of the p.Ala203Thr variant reveals complete evolutionary conservation across species. **c**) Genealogical diagram for the *WDR45B*-related CSCM in case 13. Pedigree analysis of case 13 revealed an inbreeding loop as both parents were closely related to the sire Mascol (1). See Fig. 4 for description of symbols used. **d**) Coverage plots of the sequenced affected calf and a healthy control. Note the increasing of the coverage across all the chromosome 12 (trisomy) present in the affected calf and absent in the control. Red line: genome-wide average coverage

The results of variant filtering, assuming a recessive inheritance and considering each case individually, are shown in Table 2. Filtering for homozygous variants present only in the genomes of the individual cases, using a global cohort of 5,347 bovine control genomes, allowed the identification of three private homozygous variants affecting three different candidate genes, *SHC4* in case 4 in group 1 and *KIAA0141* and *WDR45B* in cases 12 and 13, respectively, in group 3. Both sequenced parents of case 4 were heterozygous for the identified *SHC4* variant, consistent with recessive inheritance. These three identified missense variants were further analyzed for their predicted effect on the encoded protein, of which only two were predicted to be deleterious (Table 3). A homozygous missense variant in the first exon of *SHC4* (Chr10:g.61284558G > T; c.58G > T) was identified in case 4 and predicted to be deleterious because the p.Gly20Trp substitution affects a highly conserved residue in the non-cytoplasmic domain of SHC4 (Fig. 4). A homozygous missense variant in exon 7 of *WDR45B* (Chr19:g.50011776G > A; c.607G > A) was identified in case 13 and also predicted to be deleterious because the p.Ala203Thr substitution affects a highly conserved residue located in the functionally relevant proppin domain of WDR45B (Fig. 5a, b).

**Table 2:** Results of variant filtering assuming a recessive mode of inheritance as the cause of CSCM.

Group	Case ID	All variants	Private variants in the CSCM-affected calf using 1,023 cattle genome controls	Private protein changing variants in the CSCM-affected calf using 1,023 cattle genome controls	Remaining protein-changing private variants using a global control cohort of 4,540 cattle genomes and subsequent IGV inspection	Candidate gene
1	1	2,672,066	2,203	13	0	None
	2	2,705,351	792	20	0	None
	3	2,959,149	2,193	29	0	None
	4	2,829,095	2,709	14	4	*SHC4*
	5	2,941,884	1,108	5	0	None
	6	3,005,552	1,346	7	1	None
	7	2,884,699	675	4	0	None
	8	2,787,622	1,020	4	0	None
2	9	2,809,386	502	1	0	None
	10	2,789,127	430	2	0	None
	11	2,868,616	1,370	5	0	None
3	12	2,849,663	980	13	2	*KIAA0141*
	13	2,857,496	1,561	13	1	*WDR45B*
	14	2,868,462	1,065	3	0	None

**Table 3:** List of detected candidate single-nucleotide variants and small indels for CSCM in cattle

Group	Case ID	Gene	Associated phenotype in humans or gene function	Mode of inheritance	Variant type	Variant features (genomic, cDNA, protein)	Var/Var in a global control cohort of 4540 cattle genomes	Var/Ref in a global control cohort of 4540 cattle genomes	Predicted effect^#^	pLI score*
1	4	*SHC4*	Associates with receptor tyrosine kinases and has a role in acetylcholine receptor clustering at postsynaptic neuromuscular junctions; highly expressed in brain, followed by skeletal muscle (OMIM617372)	AR	Missense	Chr10: g.61284558G > T c.58G > T p.Gly20Trp	0	12 (Holstein)	Deleterious	0
3	12	*DYNC1H1*	Charcot-Marie-Tooth disease, axonal, type 2O; cortical dysplasia, complex, with other brain malformations, spinal muscular atrophy, lower extremity-predominant (AD; OMIM 600,112)	AD	Frameshift	Chr21:g.66,902,778 A > AC c.10773dupC p.Ser3592fs	0	0	Deleterious	1
	13	*WDR45B*	Neurodevelopmental disorder with spastic quadriplegia and brain abnormalities with or without seizures (AR; OMIM609226)	AR	Missense	Chr19:g.50011776G > A c.607G > A p.Ala203Thr	0	7 (Holstein and Yakut)	Deleterious	0.09

Within the control population of 1,209 Holsteins included in the 1000 Bull Genomes Project (run 9), a further 10 heterozygous *SHC4* carriers were identified. Pedigree analysis identified the Holstein sire Kinglea Leader (born 14 December 1982) as a common ancestor of the parents of case 4 (Fig. 4). The hypothesized carrier status of this sire could not be confirmed due to the unavailability of his DNA. However, one of his male offspring, Kommandeur Leader 1, was included in the 1000 Bull Genomes Project and confirmed to be heterozygous for this *SHC4* variant. Kommandeur Leader 1 was genetically related to the dam of case 4. The sire of case 4, the bull Cape P, was genetically related to Kinglea Leader through a family line that included the sire Ladino Park Talent, who was sequenced and found to be a carrier of the *SHC4* variant. The pedigree of Kinglea Leader was analysed against the pedigree of other *SHC4* carriers being available in the 1000 Bull Genomes Project cohort. This revealed that the *SHC4* heterozygous sire Bit-O-Wind Starwar was genetically related to Kinglea Leader through the sire Wapa Arlinda Conductor (born 30 August 1970) (Fig. 4). Hypothetically, Wapa Arlinda Conductor could be the founder animal for the missense variant in *SHC4*, unless it was inherited to him from one of his parents. A further four *SHC4* carriers available from the 1000 Bull Genomes Project were genetically related to the individuals shown in Fig. 4, while pedigree information was not available for two other heterozygous bulls.

Pedigree analysis of case 13 revealed an additional inbreeding loop as both parents were closely related to the sire Mascol (born 13 July 2000) (Fig. 5c). This bull had been included in the 1000 Bull Genomes Project and variant data inspection revealed that he and three of his male offspring were heterozygous for the missense variant in *WDR45B*. Three additional *WDR45B* heterozygous carriers were available from the 1000 Bull Genomes Project, but pedigree data was not available. Two of the animals were from the Yakut breed, suggesting that the deleterious mutation is also present in this breed.

### Potential de novo mutational events might explain further CSCM cases

**Fig. 6:**
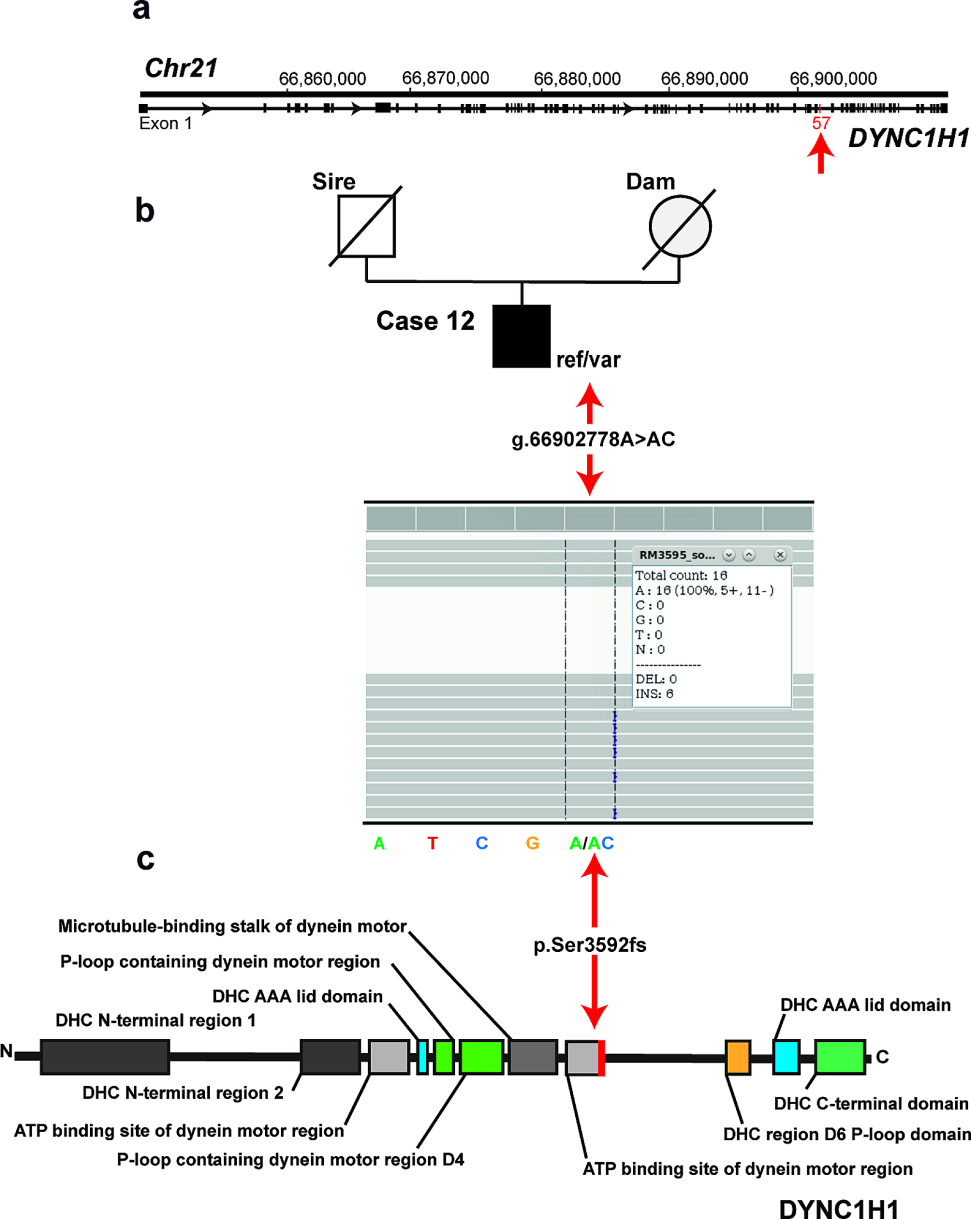
A case of CSCM with a heterozygous *DYNC1H1* frameshift variant. **a**) Case 12: *DYNC1H1* gene structure showing the location of the heterozygous frameshift insertion on chromosome 21, exon 58 (red arrow). **b**) Pedigree of the case with the genotype below the symbols and the IGV screenshot showing the Chr21: g.66,902,778 A > AC variant heterozygous in the affected calf revealed by whole-genome sequencing (shown below). **c**) The schematic representation of the bovine DYNC1H1 protein and its functional domains. The position of the p.Ser3592fs variant is indicated in red

Filtering for heterozygous private protein-changing variants, assuming that spontaneous dominant acting *de novo* mutations cause individual CSCM, WGS data were filtered for heterozygous variants present in each case individually and absent in all controls, including the parents (when available). This revealed three independent coding variants in three different candidate genes, *NOC3L* in case 7 of group 1, *SMC1B* in case 9 of group 2 and *DYNC1H1* in case 12 of group 3 (Table 4). Due to the severity of the syndrome, we hypothesized that loss-of-function variants affecting the coding sequence of a gene would most likely be responsible for CSCM. Under this assumption, only case 12 was found to represent a potentially pathogenic variant (Table 3). The heterozygous frameshift insertion in exon 57 of *DYNC1H1* (Chr21: g.66,902,778 A > AC; c.10773dupC) identified exclusively in the genome of case 12 (Fig. 6a, b), was predicted to result in a significantly truncated mutant protein (p.Ser3592fs), if expressed at all, and therefore most likely in haploinsufficiency (Fig. 6c).

**Table 4:** Results of variant filtering assuming a dominant *de novo* variant as the cause of CSCM.

Group	Case ID	All variants	Private variants in the CSCM-affected calf using 1,023 cattle genome controls	Private protein changing variants in the CSCM-affected calf using 1,023 cattle genome controls	Remaining protein-changing private variants using a global control cohort of 4,540 cattle genomes and subsequent IGV inspection	Candidate gene
1	1	5,069,395	1,008	15	1	None
	2	4,878,777	594	6	0	None
	3	4,470,944	418	1	0	None
	4	4,640,523	415	0	0	None
	5	4,481,067	330	0	0	None
	6	4,414,125	251	2	0	None
	7	4,624,854	307	2	1	*NOC3L*
	8	4,505,642	442	0	0	None
2	9	4,727,563	825	7	1	*SMC1B*
	10	4,757,074	444	0	0	None
	11	4,616,851	753	5	0	None
3	12	4,669,007	1,147	9	4	*DYNC1H1*
	13	4,713,272	1,064	10	0	None
	14	4,576,466	1,262	5	0	None

**Fig. 7:**
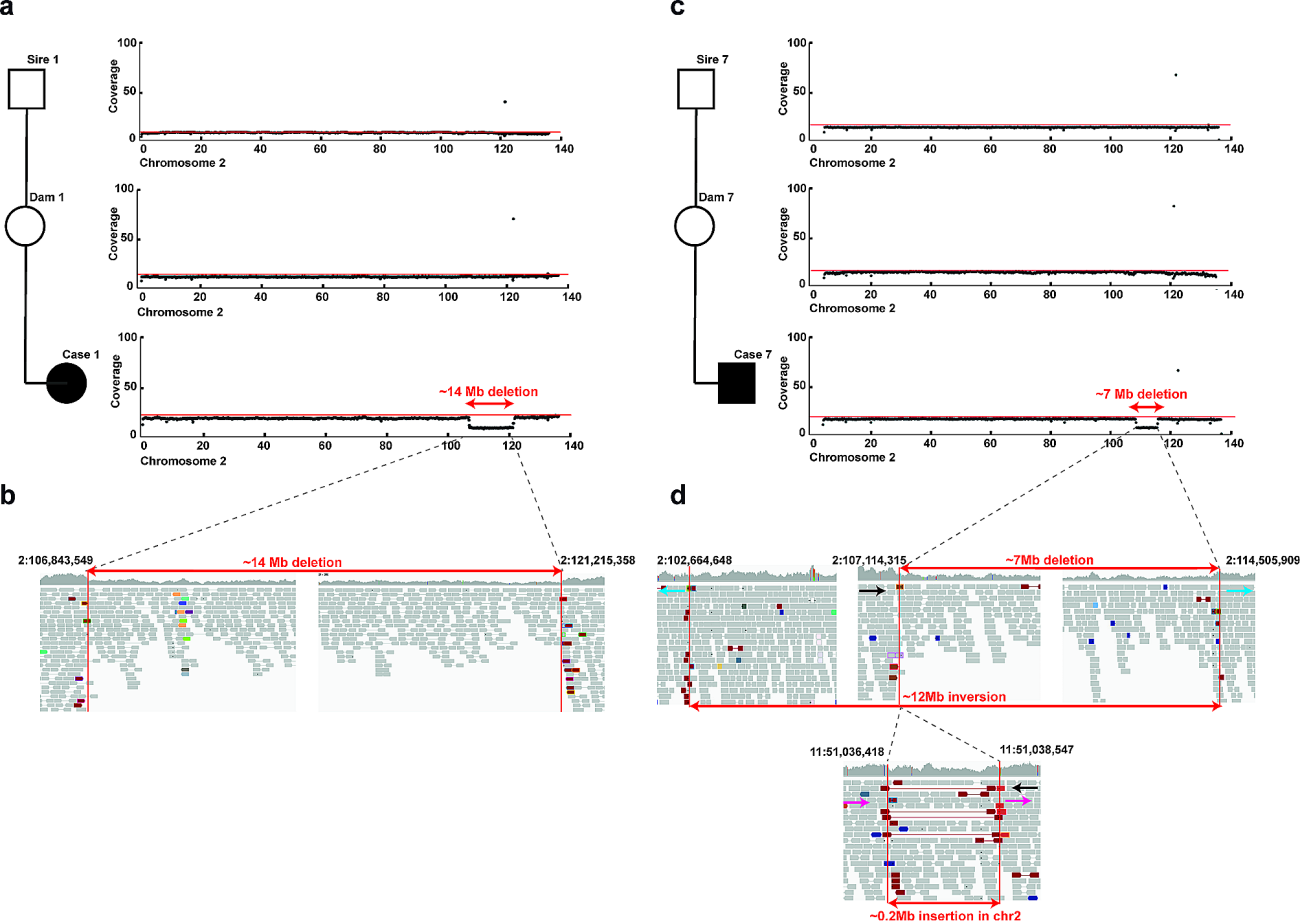
Two cases of CSCM with chromosome 2 abnormalities. **a**) Case 1: coverage plots of the sequenced affected calf, its dam and sire. Note the decrease of the coverage in the end of chromosome 2 present in the affected calf and absent in its sire and dam. Red line: genome-wide average coverage. **b**) Case 1: IGV screenshot presenting the breakpoint regions represented by a decrease on the coverage and the identified ~ 14 Mb heterozygous deletion between the two breakpoints in the affected calf. **c**) Case 7: coverage plots of the sequenced affected calf, its dam, and sire. Note the decrease of the coverage in the end of chromosome 2 present in the affected calf and absent in its sire and dam. Red line: average coverage for the 200 kb. **d**) Case 7: IGV screenshot presenting the breakpoint regions represented by a decrease on the coverage and the identified ~ 7 Mb heterozygous deletion between the two breakpoints in the affected calf. Furthermore, a ~ 12 Mb inversion of chromosome 2 is presented. The insertion of ~ 0.2 Mb of a portion of chromosome 11 is also presented

Finally, the presence of larger structural DNA variants and chromosomal abnormalities was examined in all cases. Analysis of the depth of coverage along the chromosomes revealed three observations: two different partial monosomies of chromosome 2 segments in cases 1 and 7 in group 1 with a common deleted region of ~ 7 Mb encompassing 73 coding genes (Fig. 7, Additional file 5), and a trisomy of chromosome 12 in the *WDR45B* homozygous case 13 in group 3 (Fig. 5d). For the remaining cases, no evidence of chromosomal abnormalities was found.

For case 1, visual inspection of the pair-end sequence read alignments and identification of the breakpoints confirmed the suspected heterozygous deletion on chromosome 2, which was unique to the case and absent from both the sire and dam genomes, representing a *de novo* mutation (Fig. 7a). The precise breakpoint regions were mapped by visual inspection, with the first breakpoint at Chr2:106843549 and the second breakpoint at Chr2:121215358, resulting in a 14.37 Mb deletion (Fig. 7B) encompassing 195 encoded genes, of which at least 11 (*ATG9A, EPHA4, CUL3, AGFG1, TRIP12, PSMD1, NCL, DIS3L2, ZNF362, RNF19B, S100PBP* and *RBBP4*) belong to the class of human orthologous genes with loss-of-function intolerance (Fig. 7b). The findings obtained in case 7 were consistent with a more complex structural variant including a partial monosomy of chromosome 2. Visual inspection of the pair-end sequence read alignments and identification of the breakpoints confirmed the read depth-based heterozygous deletion identified on chromosome 2, which was unique to the case and absent from both parental genomes, therefore also representing a *de novo* mutation (Fig. 7c). The exact breakpoint regions were mapped with the proximal breakpoint of the deletion on Chr2:107114315 and the distal breakpoint was on Chr2:114505909 resulting in a 7.39 Mb deletion (Fig. 7d). In addition, a partial chromosome 2 inversion of 11.84 Mb was also observed with the first breakpoint at Chr2:102664648 and the second breakpoint at Chr2:114505909 (same position as in the deletion). Visual inspection of the pair-end sequence read alignments of the first deletion breakpoint (Chr2:107114315) revealed, an additional translocation of a 0.21 Mb segment from chromosome 11 (Chr11:51036418–51,038,547) (Fig. 7d). Based on the Comparative Genome Viewer [32], bovine chromosomes 2 and 12, where chromosomal abnormalities were identified in three CSCM-affected calves, overlapped almost completely with human chromosomes 2 and 13, respectively.

## Discussion

This study was carried out on 14 calves sharing a congenital syndrome with a common lesion prototype, briefly consisting of a CMII-like lesion, spina bifida, and bilateral symmetrical hindlimb arthrogryposis. Although not all cases were necropsied, it is reasonable to consider the photo-documented cases as belonging to the same group of cases, as the morphology of all cases was similar. For the first time, genomic analysis of CSCM-affected calves showed that this syndromic congenital defect in cattle is due to extensive genetic heterogeneity, including both possible recessive alleles and dominant acting *de novo* mutations. The types of possibly causative variants associated with CSCM ranged from SNVs and small indels to larger structural DNA variants. Such a diverse spectrum of genetic anomalies may explain the variety of phenotypical presentations found at necropsy (Table 1). The finding that CSCM in cattle is likely caused either by deleterious recessive alleles in different genes or by different spontaneous mutations including chromosomal abnormalities opens up new perspectives in the understanding of CSCM and the function of the genes involved. However, a more detailed study of CSCM cases combined with further genotyping of candidate SNVs and functional experiments will be needed to better understand the association between the identified genetic variants and this congenital syndrome.

Using a trio-based WGS approach, a rare homozygous missense variant in *SHC4* was identified in a single CSCM-affected Holstein calf. Defects caused by recessive alleles represent a permanent risk to cattle breeding. However, based on our current finding of only 10 genetically closely related carriers of the highly likely pathogenic *SHC4* variant in the population of approximately 1,200 Holsteins included in the 1000 Bull Genomes Project, the impact of *SHC4*-related CSCM on the Holstein breed is expected to be limited. *SHC4* is highly expressed in the brain [34] and encodes the SHC-transforming protein 4, which is involved in the VEGF signalling and the Ras-MAPK-Erk pathway [35]. VEGF signalling plays a critical role in the migration of neural crest cells [36]. As a result, a significant number of congenital defects associated with the neural crest have been identified, potentially leading to severe craniofacial, cardiovascular, and autonomic nervous system malformations [37–39]. The MAPK-Erk pathway plays a crucial role in the regulation of a wide range of cellular processes, such as cell growth, survival, proliferation, and differentiation, in both developing and adult tissues, particularly in the brain [40, 41]. MAPK-Erk signalling components can be associated with a wide range of developmental disorders that are collectively referred to as RASopathies [40]. This group of disorders shares common features, including short stature, craniofacial and cardiac defects, and neurocognitive disabilities, all of which are often associated with abnormal brain development [42, 43]. Recently, mutations in the MAPK-ErK signalling pathway have also been reported to be associated with CM type I in humans [44]. Therefore, in addition to the rarity of the variant allele and the absence of other homozygotes, we argue that the missense variant in *SHC4* found in the single CSCM-affected calf is likely to be causal.

Using the solo-case WGS approach, we also identified a homozygous missense variant in *WDR45B* in case 13, which also had trisomy 12. It is not possible to argue whether one or even both variants actually caused the CSCM phenotype of the affected Holstein calf, so we would like to present arguments for both. Case 13 appears to have resulted from consanguineous breeding of closely related parents, which could support recessive *WDR45B*-related inheritance. The parents are genetically related through the sire Mascol, who is a heterozygous carrier of the variant, as are some of his male offsprings that were available through the 1000 Bull Genomes Project [21]. Based on this close relationship between the few heterozygous carriers identified, the impact of the *WDR45B* variant seems to be limited to recent Holstein cattle derived from Mascol, born in 2000. *WDR45B* encodes the WD repeat domain phosphoinositide-interacting protein 3, which is a key component in many biological functions, including the major intracellular degradation process by which cytoplasmic material is packaged into autophagosomes and delivered to lysosomes for degradation [45]. In humans, homozygous variants in *WDR45B* are associated with a severe neurodevelopmental disorder with spastic quadriplegia and brain abnormalities with or without seizures (OMIM 617,977). Patients with this disorder present with microcephaly, kyphoscoliosis, and multiple CNS defects [46, 47]. On the other hand, several trisomies have been reported in cattle but none have involved chromosome 12 or been related to CSCM [48]. In contrast, in human medicine, trisomy 18 has been reported to be associated with CSCM [49, 50] supporting the hypothesis that a trisomy may be associated with the development of CSCM. Furthermore, in humans, trisomy 13 (corresponding to bovine trisomy 12) is associated with a sublethal to lethal complex multiorgan malformation syndrome characterized by structural abnormalities such as holoprosencephaly, Dandy-Walker complex, cardiac defects, facial clefts, cystic hygroma, omphalocele, urinary tract abnormalities and polydactyly [51]. Therefore, it could be argued that the CSCM in the affected calf is only due to trisomy 12 and that the *WDR45B* variant may not be harmful. On the other hand, combinations of SNVs and larger structural genetic variants have previously been reported in humans with Marfan syndrome in association with mutations in *FBN1* and *SDHB* and trisomy 21 [52]. Functional studies at the protein level would be required to clarify whether *WDR45B* is implicated in bovine CSCM. In addition, genotyping of thousands of Holstein cattle and collecting more affected cases with the same homozygous *WDR45B* genotype could help to confirm or rule out the possible causality of this variant.

Finally, two independent heterozygous *de novo* structural variants involving Mb-sized segments of chromosome 2 were identified using the trio-based WGS approach. Case 1 had a partial monosomy of chromosome 2, while case 7 had a more complex structural variant that also included a partial monosomy of chromosome 2 combined with a translocation of 210 kb from chromosome 11. The common haploinsufficient region on chromosome 2 includes, among many other genes, at least three orthologs of human genes (*ATG9A*, *EPHA4*, and *CUL3*) that are classified as haploinsufficient loss-of-function genes with a probability of a loss-of-function intolerance score of 1 [31]. Recent large-scale data from human studies presented in the Developmental Disorder Genotype-Phenotype Database showed that the human *ABCB6*, *CUL3*, and *PAX3* genes, whose bovine orthologues are located in the commonly deleted genomic region, are associated with monoallelic developmental disorders [53]. Additionally, data from mouse studies of developmental disorders classified *SPEG*, also found in the shared deletion, as a lethal gene [54]. In human clinical genetics, partial monosomy of the end of the long arm of chromosome 2 (corresponding to bovine chromosome 2) is associated with the Albright’s hereditary osteodystrophy-like (AHO-like) syndrome (OMIM 600,430), which has a heterogeneous clinical presentation including brachydactyly, short stature, intellectual disability, behavioural abnormalities, and craniofacial dysmorphism [55]. However, the affected genomic region in the two bovine CSCM cases did not correspond to the previously reported causal deletion of human chromosome 2q37 [55].

Using a single case WGS approach, we were able to identify an exclusively heterozygous, ultimately rare, and therefore possibly spontaneous, frameshift insertion in *DYNC1H1* in case 12. *DYNC1H1* encodes the cytoplasmic dynein 1 heavy chain 1, which acts as a motor for the intracellular retrograde motility of vesicles and organelles along microtubules [56]. In humans, heterozygous mutations in *DYNC1H1* have been associated with neuromuscular and neurodevelopmental disorders such as spinal muscular atrophy with lower limb dominance (OMIM 158,600) [57], Charcot-Marie-Tooth disease axonal type 2O (OMIM 614,228) [58] and cortical dysplasia with other brain malformations (OMIM 614,563) [59]. Therefore, we speculate that the identified bovine loss-of-function variant causing haploinsufficiency may be causal. However, we could not confirm the *de novo* nature of this variant due to the lack of parental DNA samples.

Our WGS approach was able to provide a molecular genetic diagnosis in approximately one third of the investigated CSCM cases. The efficiency of WGS for genetic diagnosis in cattle was recently investigated for a lethal congenital syndrome in cattle with a diagnostic rate of approximately 50% [60]. Although the results obtained in the current study show a slight decrease compared to the genomic study on schistosoma reflexum [60], they are still promising when compared to the efficacy of WGS-based genetic diagnosis in humans, where putatively causal genetic variants have been reported in 25% of patients [61]. Furthermore, the lack of a possible genetic diagnosis in nine of our CSCM cases may be due to several factors, including methodological limitations such as the incomplete annotation of the bovine genome, the focus on coding variants in selected candidate genes based on literature searches, challenges associated with the short-read WGS approach, inaccuracies in read alignment and variant calling [62], or potential influences of epigenetics or non-genetic factors.

## Conclusions

This landmark study represents the first detailed genomic evaluation of bovine CSCM and reveals evidence of significant genetic heterogeneity, including different possible modes of inheritance. The results presented include independent spontaneous *de novo* mutations and recessive alleles, and different types of variants, such as SNVs, small indels, and larger structural variants including chromosomal abnormalities as potential causes of this fatal developmental disorder. For the first time, we propose candidate causal variants that may explain bovine CSCM in a certain proportion of affected calves. Finally, we provide cattle as a spontaneous large animal model for CSCM. As a result, we have not only unravelled the complexity of bovine CSCM, but also identified new potential candidate genes and variants that may have implications for related diseases in both animal and human populations. This comprehensive study advances our understanding of the genetic aetiology of bovine CSCM as a large animal model for human CMII.

ID, identification; AR, autosomal recessive; AD, autosomal dominant; OMIM, Online Mendelian Inheritance in Man database; Ref, reference allele; Var, variant allele; cDNA, complementary DNA; pLI, probability of being intolerant; Chr, chromosome; g., genomic position; c., cDNA position; p., protein position; ^#^ according to PredictSNP1 [29] and/or Provean [30]; * according to GnomAD [31].

### Electronic supplementary material

Below is the link to the electronic supplementary material.

**Additional file 1**: Sample IDs from whole-genome sequencing data stored in the European Nucleotide Archive

**Additional file 2**: Sequence accession numbers of candidate variants. All references correspond to the NCBI accessions using ARS-UCD1.2 reference genome

**Additional file 3**: Significant loci (above the Bonferroni threshold) identified in the sequencing-based genome-wide association study for the Holstein CSCM cases considering a control cohort of 166 phenotypically normal, not closely related Holstein cattle

**Additional file 4**: Sequencing-based genome-wide association study results for the Holstein CSCM cases considering a control cohort of 166 phenotypically normal, not closely related Holstein cattle

**Additional file 5**: Common deleted region of ~ 7 Mb on chromosome 2 in cases 1 and 7, encompassing 73 coding genes

## Data Availability

Whole-genome sequence data generated from the CSCM-cases and when available their parents are available under study accession PRJEB28191 and under samples accession numbers from the European Nucleotide Archive (ENA) presented in Additional file 1.

## Abbreviations

bp: Base pair
CM: Chiari malformation
CMII: Chiari malformation type II
CSCM: Congenital syndromic Chiari-like malformation
IGV: Integrative Genomics Viewer
NCBI: National Center for Biotechnology Information
OMIA: Online Mendelian Inheritance in Animals, https://omia.org/home/
pLI: Probability of a gene being intolerant
seqGWAS: Sequencing-based genome-wide association study
SNV: Single-nucleotide variant
WGS: Whole-genome sequencing
