# Supplementary material for: Congenital syndromic Chiari-like malformation (CSCM) in Holstein cattle: towards unravelling of possible genetic causes
Source: Acta Vet Scand. 2024 Jul 4;66:29. doi: 10.1186/s13028-024-00752-y (PMC11229497; doi:10.1186/s13028-024-00752-y)
Supplement: Supplementary file 1 — Additional file 1: Sample IDs from whole-genome sequencing data stored in the European Nucleotide Archive [file 13028_2024_752_MOESM1_ESM.docx]

**Additional file 1:** Sample IDs from whole-genome sequencing data stored in the European Nucleotide Archive.

| **ID** | **BAM ID** | **Sex** | **Breed** | **EBI Project ID** | **EBI Sample ID** | **Average Coverage** |
| --- | --- | --- | --- | --- | --- | --- |
| Case 1 | RM2780 | f | Danish Red Dairy x Holstein | PRJEB18113 | SAMEA6528900 | 22.4 |
| Dam of case 1 | RM2779 | f | Danish Red Dairy | PRJEB18113 | SAMEA6528899 | 16.0 |
| Sire of case 1 | RM2781 | m | Holstein | PRJEB18113 | SAMEA6528901 | 8.9 |
| Case 2 | RM3594 | m | Holstein | PRJEB18113 | SAMEA7690241 | 17.6 |
| Dam of case 2 | RM3606 | f | Holstein | PRJEB18113 | SAMEA7690253 | 17.1 |
| Sire of case 2 | RM3615 | m | Holstein | PRJEB18113 | SAMEA7690262 | 16.8 |
| Case 3 | RM3596 | m | Holstein | PRJEB18113 | SAMEA7690243 | 18.5 |
| Dam of case 3 | RM3608 | f | Holstein | PRJEB18113 | SAMEA7690255 | 18.4 |
| Sire of case 3 | RM3617 | m | Holstein | PRJEB18113 | SAMEA7690264 | 16.9 |
| Case 4 | RM3598 | m | Holstein | PRJEB18113 | SAMEA7690245 | 18.7 |
| Dam of case 4 | RM3609 | f | Holstein | PRJEB18113 | SAMEA7690256 | 17.7 |
| Sire of case 4 | RM3619 | m | Holstein | PRJEB18113 | SAMEA7690265 | 16.8 |
| Case 5 | RM3600 | m | Holstein | PRJEB18113 | SAMEA7690247 | 18.9 |
| Dam of case 5 | RM3610 | f | Holstein | PRJEB18113 | SAMEA7690257 | 17.3 |
| Sire of case 5 | RM3621 | m | Holstein | PRJEB18113 | SAMEA7690266 | 19.4 |
| Case 6 | RM3601 | m | Holstein | PRJEB18113 | SAMEA7690248 | 20.4 |
| Dam of case 6 | RM3611 | f | Holstein | PRJEB18113 | SAMEA7690258 | 21.3 |
| Sire of case 6 | RM3622 | m | Holstein | PRJEB18113 | SAMEA7690267 | 17.9 |
| Case 7 | RM3602 | f | Holstein | PRJEB18113 | SAMEA7690249 | 19.6 |
| Dam of case 7 | RM3612 | f | Holstein | PRJEB18113 | SAMEA7690259 | 17.6 |
| Sire of case 7 | RM3664 | m | Holstein | PRJEB18113 | SAMEA8565042 | 22.5 |
| Case 8 | RM3659 | m | Holstein | PRJEB18113 | SAMEA8565039 | 19.8 |
| Dam of case 8 | RM3660 | f | Holstein | PRJEB18113 | SAMEA8565040 | 22.5 |
| Sire of case 8 | RM3661 | m | Holstein | PRJEB18113 | SAMEA8565041 | 22.4 |
| Case 9 | RM3603 | m | Holstein | PRJEB18113 | SAMEA7690250 | 18.8 |
| Dam of case 9 | RM3613 | f | Holstein | PRJEB18113 | SAMEA7690260 | 18.1 |
| Case 10 | RM3605 | f | Holstein | PRJEB18113 | SAMEA7690252 | 18.0 |
| Dam of case 10 | RM3614 | f | Holstein | PRJEB18113 | SAMEA7690261 | 19.8 |
| Case 11 | RM3599 | m | Holstein | PRJEB18113 | SAMEA7690246 | 20.2 |
| Sire of case 11 | RM3620 | m | Holstein | PRJEB18113 | SAMEA8565038 | 21.2 |
| Case 12 | RM3595 | m | Holstein | PRJEB18113 | SAMEA7690242 | 19.3 |
| Case 13 | RM3597 | m | Holstein | PRJEB18113 | SAMEA7690244 | 19.8 |
| Case 14 | RM3604 | m | Holstein | PRJEB18113 | SAMEA7690251 | 17.4 |
