# Supplementary material for: Congenital syndromic Chiari-like malformation (CSCM) in Holstein cattle: towards unravelling of possible genetic causes
Source: Acta Vet Scand. 2024 Jul 4;66:29. doi: 10.1186/s13028-024-00752-y (PMC11229497; doi:10.1186/s13028-024-00752-y)
Supplement: Supplementary file 2 — Additional file 2: Sequence accession numbers of candidate variants. All references correspond to the NCBI accessions using ARS-UCD1.2 reference genome [file 13028_2024_752_MOESM2_ESM.docx]

**Additional file 2:** Sequence accession numbers of candidate variants. All references correspond to the NCBI accessions using ARS-UCD1.2 reference genome.

| **Gene** | **Chromosome** | **mRNA** | **Protein** |
| --- | --- | --- | --- |
| *SHC4* | NC_037337.1 (Chr10) | NM_001205912.1 | NP_001192841.1 |
| *WDR45B* | NC_037346.1 (Chr19) | NM_001075247.1 | NP_001068715.1 |
| *DYNC1H1* | NC_037348.1 (Chr21) | NM_001206138.1 | NP_001193067.1 |
