# Supplementary material for: Congenital syndromic Chiari-like malformation (CSCM) in Holstein cattle: towards unravelling of possible genetic causes
Source: Acta Vet Scand. 2024 Jul 4;66:29. doi: 10.1186/s13028-024-00752-y (PMC11229497; doi:10.1186/s13028-024-00752-y)
Supplement: Supplementary file 3 — Additional file 3: Significant loci (above the Bonferroni threshold) identified in the sequencing-based genome-wide association study for the Holstein CSCM cases considering a control cohort of 166 phenotypically normal, not closely related Holstein cattle [file 13028_2024_752_MOESM3_ESM.docx]

**Additional file 3:** Significant loci (above the Bonferroni threshold) identified in the sequencing-based genome-wide association study for the Holstein CSCM cases considering a control cohort of 166 phenotypically normal, not closely related Holstein cattle.

Significant loci (above the Bonferroni threshold) identified in the seqGWAS for all 13 Holstein CSCM cases:

| **Chromosome** | **SNP name** | **Position^a^** | **Allele 1** | **Allele 0** | **Allele frequency** | **beta** | **se** | **P-value** |
| --- | --- | --- | --- | --- | --- | --- | --- | --- |
| 29 | 29:45688359 | 45688359 | C | G | 0.033 | 0.618503 | 0.06542403 | 2.40357E-12 |
| 27 | 27:2638392 | 2638392 | C | T | 0.017 | 0.761923 | 0.094745 | 1.67453E-10 |
| 16 | 16:829705 | 829705 | A | G | 0.103 | 0.329078 | 0.043341 | 2.82017E-10 |
| 30 | X:105926129 | 1.06E+08 | A | G | 0.022 | 0.615518 | 0.082112 | 5.30732E-10 |
| 30 | X:32468322 | 32468322 | G | T | 0.025 | 0.597901 | 0.080154 | 5.52271E-10 |
| 6 | 6:76545141 | 76545141 | T | G | 0.033 | 0.522815 | 0.069481 | 5.54279E-10 |
| 8 | 8:22939290 | 22939290 | C | G | 0.028 | 0.538701 | 0.074467 | 5.77297E-10 |
| 16 | 16:829653 | 829653 | C | T | 0.083 | 0.337647 | 0.045681 | 7.64885E-10 |
| 2 | 2:24290484 | 24290484 | T | G | 0.019 | 0.639512 | 0.08844 | 9.27498E-10 |
| 29 | 29:17977708 | 17977708 | C | T | 0.011 | 0.919612 | 0.117176 | 1.10299E-09 |
| 29 | 29:17977716 | 17977716 | G | A | 0.011 | 0.919612 | 0.117176 | 1.10299E-09 |
| 10 | 10:66287891 | 66287891 | G | T | 0.092 | 0.312235 | 0.044677 | 2.23707E-09 |
| 29 | 29:38183308 | 38183308 | G | C | 0.042 | 0.453544 | 0.06549 | 2.30181E-09 |

^a^ in the ARS-UCD1.2 reference genome

Significant loci (above the Bonferroni threshold) identified in the seqGWAS for a subset of 11 Holstein CSCM cases, excluding the 2 cases with structural variants (cases 1 and 7):

| **Chromosome** | **SNP name** | **Position^a^** | **Allele 1** | **Allele 0** | **Allele frequency** | **beta** | **se** | **P-value** |
| --- | --- | --- | --- | --- | --- | --- | --- | --- |
| 29 | 29:45688359 | 45688359 | C | G | 0.031 | 0.607858 | 0.059729 | 1.07E-13 |
| 8 | 8:22939290 | 22939290 | C | G | 0.028 | 0.556458 | 0.065859 | 7.27E-12 |
| 30 | X:105926129 | 105926129 | A | G | 0.023 | 0.594536 | 0.07401 | 2.04E-11 |
| 23 | 2.774305556 | 2615 | T | G | 0.028 | 0.556151 | 0.065405 | 7.22E-11 |
| 16 | 16:829705 | 829705 | A | G | 0.099 | 0.30572 | 0.040377 | 1.75E-10 |
| 25 | 25:8215 | 8215 | A | G | 0.492 | -0.9288 | 0.120494 | 2.36E-10 |
| 11 | 11:92475135 | 92475135 | A | G | 0.014 | 0.747193 | 0.094346 | 2.44E-10 |
| 27 | 27:2638392 | 2638392 | C | T | 0.014 | 0.748626 | 0.094027 | 2.85E-10 |
| 21 | 21:693205 | 693205 | C | T | 0.023 | 0.592986 | 0.076734 | 2.96E-10 |
| 15 | 15:6545842 | 6545842 | G | T | 0.017 | 0.632527 | 0.089293 | 6.54E-10 |
| 21 | 21:688841 | 688841 | A | G | 0.466 | -0.46126 | 0.062346 | 9.93E-10 |

^a^ in the ARS-UCD1.2 reference genome
