# Supplementary material for: Congenital syndromic Chiari-like malformation (CSCM) in Holstein cattle: towards unravelling of possible genetic causes
Source: Acta Vet Scand. 2024 Jul 4;66:29. doi: 10.1186/s13028-024-00752-y (PMC11229497; doi:10.1186/s13028-024-00752-y)
Supplement: Supplementary file 4 — Additional file 4: Sequencing-based genome-wide association study results for the Holstein CSCM cases considering a control cohort of 166 phenotypically normal, not closely related Holstein cattle [file 13028_2024_752_MOESM4_ESM.docx]

**Additional file 4:** Sequencing-based genome-wide association study results for the Holstein CSCM cases considering a control cohort of 166 phenotypically normal, not closely related Holstein cattle. (A) Including all 13 Holstein CSCM cases. (B) Including a subset of 11 Holstein CSCM cases excluding the 2 Holstein cases with structural variants (cases 1 and 7).

**
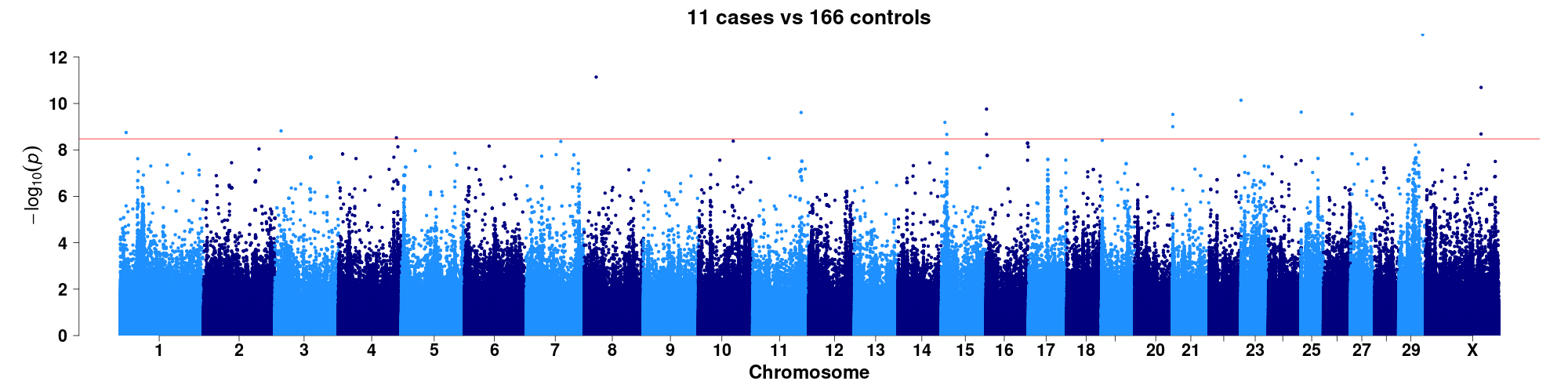
(A)
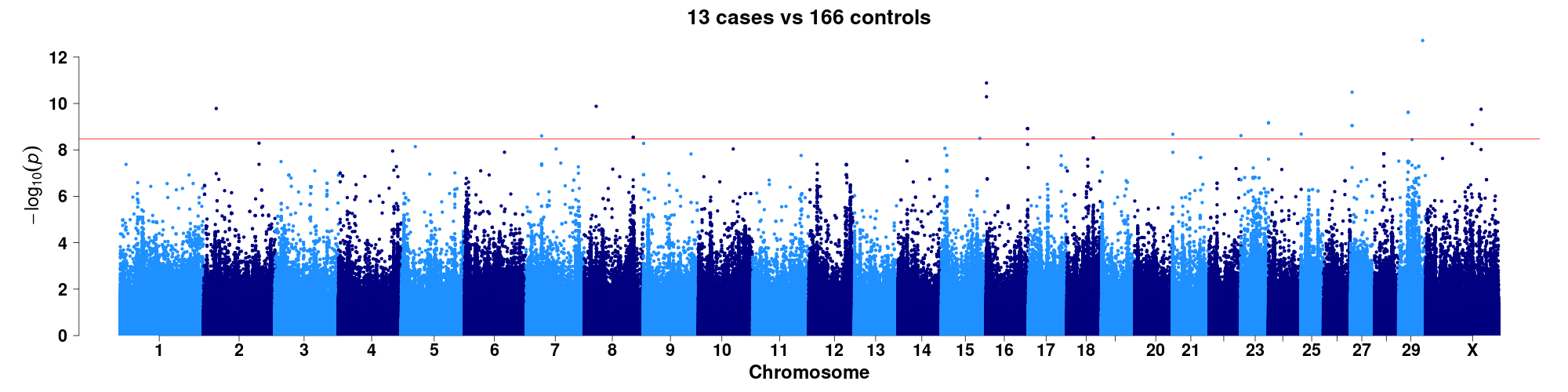
(B)**
